# Selections and Abstracts

**Published:** 1906-12

**Authors:** 


### SELECTIONS AND ABSTRACTS.

### Treatment of Acute Articular Rheumatism.*

The importance of this subject is due, not so much to the imflammatory condition, known as acute articular rheumatism, but to the deleterious after-effects on the heart. Acute articular rheumatism is invariably given as the most important etiological factor in the production of valvular heart lesions, especially in the young who might otherwise escape. So far there is no satisfactory treatment by which heart complications can be prevented unless it is prophylactic measures, which are without doubt our greatest safeguard. There can be no perfectly rational treatment for acute rheumatism until its etiology has been established. At present it is most generally believed to be a germ disease, the specific bacillus not having as yet been isolated. There are others, however, who still believe it to be due to non-neutralized acid products, the result of faulty metabolism, especially of proteids. The treatment which follows is based on the belief that both of the foregoing are etiological factors; that a condition of hyperacidity furnishes the most suitable medium for the growth and proliferation of the germ.

Prophylaxis, which may be termed the *piece de resistance* of this paper, since to prevent it is to exclude a large number of heart cases, suggests itself only in those who through heredity are supposed to have the rheumatic diathesis and in those who have had a previous attack. With such a patient the prophylactic measures are hygienic, dietetic and medicinal. He should occupy a large, airy room, which can be kept dry, a room on the second floor being preferred, since they are usually dryer than those on the first floor. Flannel should be worn next to the skin to prevent chilling from exposure to cold and dampness, either of which will precipitate an attack more quickly than

---

*Read by W. H. Anderson, M. D., Wilson, N. C., before the North Carolina Medical Society at Charlotte, N. C.

anything else, for which reason it is desirable to avoid such exposure.

The diet should be rich in certain basic constituents of food which will correct the conditions of hyperacidity and reduced alkalescence, with a minimum of animal proteid. Potassium and sodium compounds are the chief elements in maintaining alkalescence of the blood and at the same time rendering the tissue fluid alkaline, which is absolutely essential to normal metabolism. This is accomplished in a measure when the salts of these two are given as drugs, but incomparably better when they are in organic combination with vegetable proteid as provided by nature, for then they are assimilated and stored with the proteid and are present to neutralize the acids of metabolism as they are produced. The potassium compounds necessary for the human economy are taken in with foods, while the sodium compounds are furnished by the oxidation of vegetable acids. Vegetable food contains from three to four times as much potassium as animal food and furnishes vegetable acids, which by oxidation furnish the sodium compounds. The chief source of the latter, however, is from fruits, since they are especially rich in vegetable acids. Based on these facts, the diet should contain a minimum of animal proteid with a generous allowance of vegetables and fruits. Chicken, fish, oysters, bread, cereals, vegetables, fruits and milk form a suitable diet, Pastries, sweets, fried food and alcohol should be forbidden, especially the last, since it retards oxidation and so injuriously affects the liver.

Medicinal measures are usually not necessary if the proper precautions as to clothing, exposure to cold, and diet are faithfully carried out. However, it is a good precautionary measure to frequently test the patient's urine for hyperacidity, which is usually a fair index of his condition. This can usually be corrected by the use of the various alkaline mineral waters, or by giving drachm doses of a solution containing five grains each of the acetate, bicarbonate and citrate of potash to the drachm. If in addition to this the patient drinks a sufficient quantity of water the kidneys are kept in a state of activity. The bowels should be kept open with epsom salts or sodium phosphate, for it is impossible to overestimate the importance of keeping the organs of elimination in the best possible condition. And, finally, that household remedy, bicarbonate of soda, is never amiss in any stage during the treatment of rheumatism, helping as it does to maintain that alkaline condition of the secretions so desirable in this disease.

These in general are the prophylactic measures to be used and if successful the chief source of dangerous heart lesions will be eliminated.

The treatment of an acute attack of rheumatism is practically agreed upon. The patient is put to bed in a large, dry, sunny room. He is placed between woolen blankets and dressed in a light flannel nightgown with open sleeves, which may be pinned with safety-pins. These measures are useful to prevent the chilling and discomfort following the drenching acid sweats characteristic of the disease. The affected joint is wrapped in cotton batting, and if desired a splint applied. Should these be not well borne, hot fomentations are applied locally, or a solution of menthol, chloral, alcohol and camphor water used.

Free purgation is secured by calomel followed by epsom salts. The bowels are kept open throughout the course of the disease by broken doses of salts, a teaspoonful of the saturated solution being given often enough to get from three to five movements a day. After the first few days, however, if the acute symptoms are subsiding, such free purgation is usually not necessary.

Pain, which is by far the most distressing symptom, is controlled by the salicylate of soda. At times during the initial stage it is necessary to give one-eighth grain doses of morphia hypodermatically for temporary relief until the salicylate can be administered in sufficient quantities to afford more lasting results. The salicylate in ten to fifteen grain doses is given with a glass of alkaline mineral water every two hours until the pain is under control. As soon as this happy result is secured the dose is decreased and the intervals at which it is given are lengthened. If the intestinal tract has been emptied and all the organs of elimination are performing their duties well, the probability of gastric irritation will be greatly lessened. However, this may be obviated at times by giving the drug with a half glass of milk to which carbonated water has been added. Since the sole action of the salicylate is to relieve pain, it is necessary to give ten-grain doses of sodium bicarbonate to combat the condition of hyperacidity. At the same time alkaline mineral water is given *ad libitum*, and should these not prove sufficient to render the urine alkaline, or neutral, five grain doses of the acetate, bicarbonate and citrate of potassium in solution are given. Throughout the course of the disease the urine should be kept alkaline or neutral if possible.

The fever is allowed to take care of itself, since as a general thing it is not high and if it does not persist. Should hyperpyrexia be present it is treated in the usual way by sponging, cold baths or cold packs.

During the acute stage of the disease milk is the sole article of diet and even this should be withheld until the alimentary tract has been emptied and cleansed. Lemonade and orange juice may be freely given, and after the acute symptoms have subsided, vegetable soups and fruits are allowed. As soon as convalescence is established, vegetable soups, cereals, green vegetables and fruits are given, which diet after a few days, if there is no sign of relapse, may be increased by addition of fish, chicken and oysters. The return to a full proteid diet should be very gradual and discontinued at the first sign of relapse. Where the patient is not sufficiently well nourished on a non-proteid diet, it is necessary to increase the amount of proteid earlier after the acute symptoms have subsided. Throughout convalescence the patient's bowels are kept open by small, repeated doses of a saturated solution of epsom salts and the urine is kept neutral or alkaline by alkaline mineral water.

Usually a profound anemia follows an attack of acute articular rheumatism, which necessitates the use of some preparation of iron. Blaud's pills are satisfactory in adults, while the syrup of the iodide of iron is used in children, and if at the same time

Fowler's solution is given the damage to the red blood cells is repaired and recovery materially hastened.

To insure against a relapse and to obtain the speediest return to a state of health, it is best to keep the patient in bed at least a week after the acute symptoms have subsided and the temperature is normal. The greatest precaution should be taken against exposure to cold and dampness, and this same vigilance should be maintained for weeks after an apparent cure is well established.

The stethoscope should be constantly used during an attack of acute rheumatism, whether the attack is severe or mild and at the very first sign of cardiac distress prompt measures should be taken to employed relief. The necessity for this is apparent when it is remembered that at times, although the rheumatic symptoms are obscure and insidious the cardiac lesions following may be most extensive, entailing upon the patient an existence of discomfort and despair, while the futility of a hopeful prognosis will be ever present to disturb the mind of the attending physician. To know that in a great many instances the possibility of such a state of affairs may be avoided by the prevention of rheumatism, is to recognize the fact that, after all has been said, prophylactic measures are the most important in the treatment of acute articular rheumatism.—*Charlotte Medical Journal.*

---

### OBSTETICAL PROGRESS.

The July number of the *American Journal of Obstetrics* contains a number of good articles on some of the most important obstetrical problems. Broadhead gives an exhaustive study of the treatment of the toxemia of pregnancy. He declares that at the present time we believe that the toxemia of pregnancy depends on a disturbance of nitrogenous metabolism—a very concise statement of a most obscure condition, but who can improve upon it?

Sometime ago we were all testing for the quantity of urea in urine, as it had been decided that puerperal eclampsia was inaugurated by a diminution in the quantity of urea and failing kidney function. Now it is different. To quote:

"Ewing believes that the systematic study of the urine will show that unoxidized proteid derivatives are invariably present in comparatively early stages of the severer cases. Instead of urea, uric acid, ammonia, leucin, tyrosin and other unoxidized proteid radicals appear in the urine, and instead of sulphates there are unoxidized sulphur compounds. As leucin, tyrosin and ammonia are estimated with urea by the hypobromite method, the latter is unreliable."

Now this study of the urine has grown to very large proportions. It has been found that the liver does not do its work well; the nitrogen instead of being excreted as urea is transformed into ammonia. Williams states that:

"Normally from 3 to 5 per cent. of the excreted nitrogen is in the form of ammonia, whereas in the pernicious vomiting of pregnancy the amount of nitrogen put out as ammonia, as compared to the total nitrogen of the urine, may be as high as 30 to 46 per cent. In other words, the large amounts of nitrogenous materials escape oxidation and conversion into urea. According to this authority, a marked increase in the ammonia coefficient in pernicious vomiting indicates the emptying of the uterus; and he gives 10 per cent. as the danger-signal. He also states that while, in pernicious vomiting, the total nitrogen excretion is approximately normal, in eclampsia it is diminished. On the other hand, in pernicious vomiting, the ammonia coefficient is wonderfully elevated, while in eclampsia the ammonia coefficient is the same."

But this is not all—acetone and diacetic acid have been found, especially in hyperemesis gravidarum. Betaoxybutyric acid has also been discovered, so that the evidence of the toxemia is strong.

In the treatment of the toxemia of pregnancy the author gives nothing new. In the vomiting, sodium bicarbonate should be tried in large doses; its use in other forms of toxemia is also indicated.

His treatment for eclampsia is as follows:

"During the eclamptic seizure administer chloroform and oxygen if possible. Prevent the patient from biting her tongue, and from injuring herself by blows and falls. If the pulse is full and strong, with tension, give the Squibb fluid extract of veratrum viride, mx–xx, hypodermically, and repeat in doses of mv every half hour until the pulse is reduced to 60. If the Norwood's tincture is used, give mx–xx and repeat in doses of mx every half hour until the pulse is reduced to 60. In case of collapse, use whisky, morphin and atrophin hypodermatically. If the pulse is weak and feeble, rely chiefly on chloral and bromids by rectum in doses of ʒss–j of each. Where the pulse is strong use veratrum viride as indicated, combined with chloral by rectum.

"If the patient is unconscious, move the bowels by croton oil mj–ij, given with olive oil ʒj on the back of the tongue. If this is not sufficient, give a high enema of sulphate of magnesia and castor oil, of each ℥j. If the patient is conscious, give sulphate of magnesia ʒij every fifteen minutes until ℥j has been given. Then, if necessary, use the high enema of magnesia sulphate and castor oil. A hot pack should then be given. The colon should be irrigated with several gallons of saline solution and several quarts left to absorb. Intravenous infusion should be reserved for the very severe cases. Venesection, when labor has not yet begun, may be used to advantage in robust patients with a full pulse, 12 to 16 ounces of blood being removed. If, however, the patient is about to be delivered, a moderate blood loss can be allowed in the third stage, and if necessary, venesection can be performed after delivery. To decrease arterial tension, and as a heart stimulant, diuretic and diaphoretic, nitroglycerin is also of great value, while caffeine and strophanthus are second only to nitroglycerin.

"If labor has not commenced, a modified Champetier de Ribes bag should be introduced and the cervix should be softened and dilated by the use of these bags. When the cervix has been well dilated, complete dilatation by the hand, and delivery by forceps or version.

"If labor has alreedy begun, but the cervix is long and rigid, use the bag for softening and dilatation. If the cervix is soft and dilatable, complete dilatation manually and deliver by forceps or version.

"If the cervix can not be dilated by the ordinary methods, Dührssen's incisions or Cesarean section should be advised, but unless the operator feels perfectly able to perform these operations, it would be better, we believe, to rely on medical treatment alone.

"The one fact above all to keep in mind at all times is, that in elimination lies the hope of the patient's salvation."

Watkins gives the following rules for the prevention of puerperal sepsis:

"We should be surgically clean ourselves, as should everything that is to come into contact with the patient. Make the whole procedure a surgical one, and impress the patient and family of its import. Dispense with the so-called cleansing douche before labor, unless in the face of necessity, and then give it with care.

"Wear rubber gloves. You can boil the gloves; you can not boil your hands. Some say gloves are cumbersome; that is not so if they fit.

"After the birth of the child, do not make a vaginal examination unless there is absolute reason for it.

"Suture any perineal wound, unless weakness of the patient or extensive edema contraindicates. Wounds of the cervix, unless compelled by hemorrhage, are better done a little later.

"In applying the forceps remember that it is to be used as an aid to nature, not as an instrument designed to drag a child into the world in spite of any obstacle that may exist.

"Make as few vaginal examinations as possible. Careful abdominal palpation is better, and there is no danger of infecting the patient.

"Do not allow the child to be forced between a loaded bladder and rectum.

"Have the patient enter the labor in as good physical condition as possible, and it goes without saying that if sutures have been taken for any reason and have suppurated, they should be removed.

"When it can be done, have a bacteriological examination of the lochia made, but bear in mind that the clinical signs, both subjective and objective, are important.

"If the lochia is foul, profuse and frothy, examine the cavity of the uterus with your finger. The wall will usually be rough, and the cavity filled with blood clots, secundines or some debris that should be removed. Preferably this removal should be done by the finger, but if the curette is used, great care should be exercised not to destroy the granulation zone, or what is often done, puncture the uterus. Remove the little bits that the finger has broken up by a gentle saline douche, and give the patient a general supporting treatment.

"If the symptoms are those of true sepsis, the above mentioned treatment is not to be used. Above all things do not curette; it is no less than criminal. Not only is the incomplete protective zone destroyed, but the infection is spread. A great deal has been said pro and con as regards the use of the curette in this condition. If a man should say that if one was cold he should remove his overcoat and thus keep the cold out, we would not doubt the mental obliquity of that person. To my mind, it is about as much common sense to advise one to remove the protection that nature is trying to put on to keep out infection. The round cells are of value; let them alone."

We must append his rules which he has formulated, how to kill a woman suffering from sepsis:

"1. Use all the salts that you can persuade her to swallow.

"2. Poison her by strychnine.

"3. Use the curette and intrauterine douche indiscriminately.

"4. If she is still alive, perform an abdominal section."

Hill gives the statistics of 1,000 cases of labor. The figures are very instructive. A maternal mortality of 2 in 1,000 is certainly low for tenement obstetrics: quite a change this is from the time of Semelweiss. Only 18 cases of septic infection occurred. Which shows that the filth of the tenement house is by no means so septic. In fact, better results can be obtained in such homes, if care is used, than in the obstetric hospital.—*Courier of Medicine.*

---

### ADVICE TO THE BEGINNER—THE ASEPTIC LABOR.

With the advent of asepsis and the change from antiseptic methods to those of simple cleanliness, its influence became apparent in the work of the obstetrician. In private practice, especially in poor work, the problem which confronts the obstetrician is one of many perplexities, and it has occurred to every worker in this field to find that his carefully laid down antiseptic plans have been most glaringly disregarded, as it is not at all uncommon to find an inquisitive member of the family carefully inspecting, with dirty hands, the sterilized vulva pads to see what possible use they can be made for, or to find a very zealous assistant, a member of the family, testing the temperature of sterilized water with a hand the farthest possible removed from clean. These minor tragedies that rack the aseptic conscience of the operator are so numerous that it is hardly worth while to enumerate them. The question arises, is it possible to carry on a labor in a tenement house in a practically clean manner? The answer is unquestionably, yes, provided the obstetrician will be emphatic enough to make his ideas seem of real importance to the patient and her family. Little can be hoped from the monthly nurse, whose mind is usually sluggish, and whose fingers are alternately used for aseptic dressings and the preparations of the meals for the patient. In the first place, when labor is started, if it is within the range of possibility, the patient may be made to take a bath, empty the lower bowel with an enema, and scrub the external genitalia with soap and water, preferably, if the objections are not too strong, shave the parts. This will often have to be done by the obstetrician himself. Then an antiseptic vulva pad should be placed on the patient, if for no other reason, to prevent the patient investigating her own anatomy. It is important to warn the patient gravely as to the danger of her own fingers; also to warn the monthly nurse against any investigation on her part. These warnings should be given, not with gentleness, but with firmness bordering on severity.

The preparation of the bed for the patient should be made under the direction of the operator, and along the usual lines. If there is another bed available, the patient should be allowed to pass the hours of the first stage and the beginning of the second on a bed that is not to be used for the delivery, or better still a table may be used for the act of delivery. The patient's covering at the time of delivery is best made of a night-dress and a pair of pillow-cases covering feet and legs. These pillow-cases may be easily held in place by strips of adhesive plaster run around them and attaching them to the thighs, but it is useless to put them in position until after the patient has passed over the walking around stage and can at least be kept relatively quiet. A little care in the preparation of boiled cold water, absorbent cotton and plain gauze will help materially in the work that is before the obstetrician. If the family can be impressed with the importance of having the water boiled, and put into clean containers for the use of the doctor alone, or under his direction, one danger, that of using dirty water, will be avoided. Of course the boiling water used to heat the cold sterile water is free from danger. Vulva pads made from the sterile gauze, wrapped around a piece of sterile cotton, wrapped in a clean towel and placed in a bureau drawer which has been lined with a clean towel, and opened only by either the nurse or the doctor, will help. In most cases it is possible to have clean sheets for the bed if arrangements are made by the physician with the family, prior to the time of delivery. These sheets should be rendered sterile by steaming in a vessel placed inside of the ordinary clothes boiler, and above the water line, and not ironed after taking from the boiler. This method of sterilization is simple and may be applied to everything that comes in contact with the patient. If the doctor has in his obstetrical outfit a large Kelly pad for placing over the bed or on the table at the time of delivery, much of the unpleasant soiling will be averted.

The use of one post-partem douche as a matter of comfort and cleanliness is most satisfactory. The best solution is normal salt, which can be made by the boiling of ordinary table salt in a little water, adding four heaping teaspoonfuls of salt to say four ounces of water, which when added to four quarts of hot water, will make a suitable douche. This will be found very safe and comforting to the patient and will wash out any clots that may collect in the vaginal canal.

After the doctor has delivered the patient, and the patient is left clean, the one to be in charge of the patient afterward should be instructed, with severity, to avoid handling of the external parts, no matter how carefully she thinks she knows how to clean her hands. The cleansing of the external genitalia may easily be accomplished by a solution of 1 to 4,000 bichloride of mercury, the patient to be placed upon a bed-pan and the solution poured on the patient from a pitcher. Adherent particles may be washed off by gauze or cotton in the hands of the nurse with comparative safety, careful instruction being given her to wash from before backwards.

The use of rubber gloves by the accoucheur, I am aware, is a mooted question, but if the operator becomes accustomed to them he will find that they are of such advantage in the case of cleansing, and as from the very nature of the case, his hands or gloves are liable to become frequently soiled, this is a point not to be neglected.

The wearing of the sterile gown by the accoucheur, even in tenement-house practice, is very satisfactory to the operator in the saving of his own clothes; also eliminates one possible source of infection either to the instruments or to the doctor's hands. The writer is aware that these methods of the hospital are, perhaps, a little trouble to the operator in his private practice, and may impress the patient with the seriousness of the act of labor; still their importance is very great and it is surely worth while to surround the woman in labor with every possible safeguard.

If the urine has been carefully watched during the months of pregnancy, and the pelvis is normal, the patient clean, the operator clean and skillful, the act of parturition loses most of its danger and to a great extent is robbed of future ill effects. These precautions will increase the cost of labor slightly to both the patient and her physician, and to the busy doctor will cost him some time and considerable forethought, but are well worth both the expense, the trouble and the thought in the increased safety and the future benefit to the patient. Have the patient prepared for her labor, be prepared yourself, and have everything in readiness for what will be to her a severe ordeal and what may tax your skill to the utmost.—*Ed. Annals Gyn. and Pediatry, October, 1906.*

---

### PLAGUE INVESTIGATIONS IN INDIA.

The commission appointed by the government to investigate plague in India has issued a highly important series of reports. Apart from the comparatively rare pneumonic form, ordinary plague is found not to be particularly infectious, and man-to-man infection plays no important part in the spread of epidemics. Plague, nevertheless, may be introduced into a new locality by the agency of man and the agent need not be a sufferer from the disease. Definite localities, such as huts or rooms, in which plague has occurred among rats and men during an epidemic, are highly infectious. Persons living or sleeping in such places are liable to contract the disease, although similar close attendance on a plague case in a hospital or temporary camp is seldom dangerous. The infection clings to the described localities, and with such tenacity that persons returning to them a month, or in some cases as much as forty-eight days, after they have been evacuated have promptly contracted the disease. Plague can exist and spread under a great variety of climatic conditions, but when once it has become endemic it exhibits a marked seasonal prevalence. Bombay, for instance, is never free from plague, but the incidence is relatively small from June to December. In January and February the number of cases rises, at first slowly, then rapidly, and reaches a maximum in March and April, to fall again rapidly in the latter half of April and in May. During the first-named period the number of cases a month may be from 50 to 500, but during the height of the epidemics it may range from 4,000 to 7,000. In other parts of India the yearly outbreaks have shown similar periods of prevalence during particular months, with complete or almost complete disappearance at other times, but the months of prevalence in different localities are not always the same. In the great majority of instances the outbreak of plague among men is associated with and generally preceded by, an epidemic among rats. There are the strongest reasons for regarding the epizootic as being the most important cause of the epidemic spread of the disease. As to the part played by the flea in conveying the disease from rats to man, most important experiments have been made which include a careful anatomic examination of the suctorial apparatus of the flea. It appears that the insect, after making and enlarging its skin puncture, injects its saliva into the wound through one channel of its proboscis and withdraws blood through another. As in the case of the leech, the injection of saliva presumably prevents coagulation of the blood with which it mingles, and may afford a means of injecting the bacilli of plague, although these have not yet been detected in the salivary glands. The rat flea of India, unlike that of Europe, feeds readily on human beings. Guinea-pigs allowed to run free in plague-infected houses attracted a large number of fleas, mostly rat fleas, and twenty-nine per cent. of them died of plague. The same results followed in houses which had been disinfected in the ordinary manner. Fleas transferred from plague-infected rats found dead or dying in houses transmitted plague to healthy animals. Caged animals were placed in plague-houses in pairs, completely protected from soil and contact infection, and equally exposed to aerial infection, but one of each pair was protected from fleas and the other not. None of the protected animals contracted plague and several of the unprotected ones died from it. Among the fleas arrested and caught by the protective apparatus a certain proportion were found to contain in their stomachs abundant bacilli identical with plague bacilli. Another series of experiments showed that plague bacilli suffered no diminution of virulence in passing through the bodies of several successive rats, but that many of the Bombay rats are, comparatively at least, insusceptible to the plague virus, possibly because they have recovered from the disease. The blood of infected rats was found to contain an enormous number of plague bacilli, as many as 100,000,000 per c.c. being found before death. Finally, the existence of what is described as chronic plague was demonstrated in rats caught in previously infected localities during periods of general subsidence of the disease, showing that plague in an unsuspected form may linger in the rat population and may again spring into virulence among them under the operation of unknown causes. The whole of the experimental work appears to have been conducted with great skill. Evidently the government of India is sparing no effort to cope with the disease.—*London Letter*, *Jour. American Med. Association*, *November 10*, *1906*.

---

### VITAL POINTS IN THE TECHNIC OF SUPRAPUBIC ENUCLEATION OF THE PROSTATE FOR BENIGN ENLARGEMENT OF THAT GLAND.

E. Hurry Fenwick, London, *Journal of the American Medical Association*, October 13, 1906, says: "Our present technic in suprapubic prostatic enucleation tends (1) to the destruction of the vesical orificial ring; (2) to the wholesale destruction of the prostatic urethra with its afferent seminal ducts; (3) to the rough handling of the membranous urethra. No matter how the operation is carried out, the original vesical orifice should be left intact and covered with its own mucous membrane. A neglect of this rule in a certain proportion of cases will leave the patient with a warped or narrowed vesical orifice and its attendant evils. Unless there is enough intravesical projection to afford spare mucous membrane to replace that destroyed, such will be the case. To avoid this the author has successfully grafted in portions of a sheep's urethra, and reports a case in which this was done. He suggests that if a medium or large projecting lateral lobe is present, that it be separately enucleated by an antero-posterior incision, and that the rest of the prostate be removed by an operation described by him in 1904, in which he starts the separation from the prostatic urethra. The forefinger is inserted into the prostatic urethra up to the first joint, the point of the finger is then bent and plunged sideways through the mucous membrane, which in the soft elastic prostate gives readily before the pressure. At once the finger finds itself between the tough capsule of the prostate and the contained adenomatous masses; traveling on without much opposition, the entire lobe is enucleated and generally stripped off the urethra. Great care is taken to keep the floor of the urethra intact and attached to its bed. Usually the adhesions of the lateral walls of the urethra and the lateral lobe are very dense; that part of the canal comes away with the lobe, but the floor is preserved. The lobe is now gently detached from the triangular ligament, so as not to tear or bruise the membranous urethra, and being free, it is pushed or pulled into the bladder; the opposite lobe is treated in a similar way. The finger finally smooths down the mucous membrane in the prostatic urethra, leaving the vesical opening clear and free from projecting tags. The vesical opening heals by being lined with part of the original prostatic urethra. Fenwick emphasizes the importance of not destroying the ejaculatory ducts, and also of not injuring the membranous urethra in separating the anterior face of the prostate from the face of the triangular ligament. As this is the future true sphincter of the bladder after prostatic enucleation, it should be very gently and cautiously handled."

---

### SIMPLE ELIXIR AS A VEHICLE IN PRESCRIPTIONS INTENDED FOR CHILDREN.

By EDGAR F. HEFFNER, PH. G., Lock Haven, Pa.

Prescriptions coming to me to be filled and calling for simple elixir as a vehicle, or in considerable quantity, which prescriptions I knew to be for children of tender years, led me to investigate the physician's idea of this preparation and the quantity of alcohol it contained.

It astonished me to find occasionally a physician who thought it contained no alcohol, while the majority were of the opinion that simple elixir, and the medicinal elixirs of which it forms the basis, elixir calasaya, elixir lactopeptine, elixir gentian, etc., were of a very slight alcoholic strength. Prescriptions have come under my observation, in my present location, and in other localities, like the following:

I.

*For a child four months old:*

Sodii bromidi........................................gr. viij
Elix. simplicis.........................................oz. i
Sig: Teaspoonful every half hour or one hour as needed.

II.

*For a six-year-old child:*

Potass. bromidi........................................gr. xlviij
Elix. val. ammon........................................dram iv
Elix. simplicis, q. s........................................oz. iij
Sig: One or two teaspoonfuls in water every one or two hours as needed.

No physician would give a child four months old a quarter teaspoonful of alcohol every half hour, yet that is what the child would have been given had the prescription been filled as written.

Compared with beer it would have been the same as giving a tablespoonful, or been equal to two teaspoonfuls of wine, or over half a teaspoonful of whisky or brandy.

Prescription No. 2 shows the same thing in a more frequently used form—the six-year-old child would get with each two or four grains of bromide the equivalent in strength of one-half or one whole teaspoonful of whisky, which would effectively counteract the sedative effect of the bromide.

These examples need no extended comment as to the possible injurious effect of the alcohol on children, as we all fully appreciate them. It impresses on our minds the fact, however, that we must occasionally refresh the minds of our medical friends in cases of this nature, and the information, if given in the proper spirit, will be thankfully received by any physician needing it.

In fact, it is a question in my mind if the very common practice of giving bromides and similar sedatives in simple elixir is a good one, as the effect of the bromide is seriously interfered with by the alcohol it contains.

While on the subject of simple elixir and bromides, I wish to present an example of a prescription fairly common in some localities. It is as follows:

| | |
|---|---|
| Sodium bromide | dram iv |
| Chloral hydrate | dram iv |
| Simple elixir. q. s. | oz. iv |

Sig: Two teaspoonfuls in water every half to one hour.

This at first makes a bright clear solution and the chemical incompatibility of chloral and alkali bromides in alcoholic solution would be generally overlooked. On standing half an hour the chloral alcoholate will come to the top in a clear layer about one-fourth of an inch thick, and of a color very closely resembling the rest of the prescription. I know of one case in which the mixture was dispensed without a "Shake" label, and the patient got nearly all of the chloral in the form of alcoholate at the second dose.

The use of an aromatic water and simple syrup as a vehicle in prescriptions of this nature would obviate any danger.—*Reprint from proceedings twenty-ninth annual meeting Pennsylvania Pharmaceutical Association.*

---

### A HISTOLOGICAL STUDY OF THE KIDNEY IN SCARLATINA.*

This examination of the kidneys of patients dying during an attack of scarlet fever has been undertaken with a view of ascertaining the pathological alterations found at different stages of the fever, and of finding out how far some of the clinical features can be accounted for by the pathological changes. The result of this investigation has been to establish that the classification of the cases by their clinical features coincides with certain main types of the pathological change. Three classes are distinguished.

CLASS I. *Cases with a Definite History of Acute Nephritis Commencing about the Third Week.*—In these the pathological

*E. Seymour Chapman, M.D., in *Journal of Pathology and Bacteriology*, vol. ii, 1906.

changes were always well marked and were most characteristic. They primarily affected the Malpighian corpuscles, and consisted of proliferative and degenerative changes of the glomerular capillaries of the afferent arteries, and of the cells in Bowman's capsules. The changes affecting the tubules, on the other hand, were less characteristic, and were largely secondary to the alterations in the Malpighian corpuscles. These tubular changes were granular degeneration, and more rarely, disintegration of the cellular protoplasm. In connection with the primary changes, it was shown that all, if sufficiently advanced, would cause a diminution in the quantity of urine excreted, and this deduction was fully borne out by reference to the clinical histories of the cases. It was found that albuminuria is most ascribable to the changes in the Malpighian corpuscles, and that hematuria arises from direct hemorrhage into the tubules. Tube casts, both in process of formation and in the fully formed state, were most frequently found in the junctional tubules; and it is pointed out that all the tube casts reaching the urine were most probably formed in the smaller junctional tubules, that their granular portions resulted from the disintegration of the cells of the convoluted tubules and ascending loops of Henle, and that their epithelial elements were derived from the desquamated cells of the latter structures. A glomerular nephritis is typical of Class I.

CLASS II. *Cases in which Death Occurred within a Few Days of the Onset of the Fever.*—In these the pathological changes were neither so marked nor so characteristic, and were chiefly of a degenerative character. They consisted of granular and hyaline degeneration of the tubular epithelium, of proliferation of the cells in the ascending limb of Henle, and of hyaline changes in the smaller blood vessels. Of these changes, special reference is made to the hyaline degeneration of the tubules, to the subsequent disintegration of the cell with liberation of the hyaline globules into the interiors of the tubules and into the interstitial tissue, and also to the proliferation of the cells in the ascending portions of Henle's loop. A degenerative nephritis is typical of Class II.

CLASS III. *Cases in which Death Occurred After a Period of Six Days or More from the Commencement of the Fever.*—In these the morbid changes consisted of an infiltration of the intestinal tissue, together with a high degree of tubular degeneration. The infiltrating cells, known as plasma cells, were found to be uninuclear leucocytes in an altered form. They did not show any evidence of mitotic division or any tendency towards phagocytosis. Many degenerating tubular epithelial cells, however, were seen in the areas of infiltration, and these cells occasionally exhibited phagocytic properties. The amount of tubular degeneration corresponded with the degree of the cellular infiltration. It was found that the infiltration varied in its intensity according to the duration of the fever, that it was never present before the sixth day, and that it was evident from that day onwards in all cases except three. An acute interstitial nephritis is typical of Class III.

It is thus seen that although most of the structures in the kidney may be affected during an attack of scarlet fever the pathological changes present are definitely related to certain clinical phenomena and occur at certain definite stages in the course of the disease. First, there is a glomerular nephritis occurring in cases which during life showed unequivocal signs of acute nephritis; secondly, there is a degenerative nephritis found in very severe cases, in which death took place within a few days of the onset of the illness; and lastly, there is an acute interstitial nephritis associated with severe cases which the patient survived for a longer period than six days from the commencement of the fever.—*St. Louis Medical Review, November 10, 1906.*

---

### WHAT IS THE TRUE CAUSE OF SYPHILIS?

As we foreshadowed in a preceding editorial, the premature rejoicing of syphilologists over the discovery of Schaudinn has not borne the fruit which was so sanguinely expected. The unbelieving workers have been busy, and their work has been placed in evidence by them. Their activity may be looked upon as pernicious by those who are determined to make the spirochæta pallida the active cause of syphilis. The fight, up to the present, is a very pretty one, and is becoming invested with much interest. If we were to believe those who are opposed to the views of Schaudinn, the spirochæta pallida is not a specific bacterium, but rather an accident. The flagellæ will no doubt be held up as artefacts, or as the creatures of overheated imaginations, influenced by a misdirected enthusiasm. We have not grown enthusiastic over the discovery of the spirochæta pallida, but have ever tried to keep a conservative attitude in the matter, as it portended to be one that would justify such a position. We are now more inclined than ever to keep that position, more especially as in the light of recent events it seems to be the only one which is consistent to assume by him who looks upon and considers the matter from a purely scientific point of view. The latest to which we have been treated is the following, culled from a late issue of the *Medical Review*: "The causative factor of syphilis has long formed a topic of entertaining, even if bitter, discussion, though it was thought that the end had already been reached with the discovery of the spirochæta pallida. The evidence of the infectious character of the blood during the secondary stages of syphilis seems fairly well established, and has been practically admitted by most syphilologists; but whatever the infectious principle may be, it is essential that it be recoverable from the blood during the secondary stage of the disease. W. E. de Korte, of London, claims, in the *Practitioner* for June, 1906, that spirochæta pallida fails to satisfy this essential condition, and on no occasion in the blood of a large number of syphilitics which he examined did he once meet with this organism. He did, however, find another series of bodies which he also believes are protozoan in nature, but of their casual relationship to syphilis, he thinks it premature to speak. He observed a number of stages of this organism, which he has grouped into a life circle, and of these he has obtained photographs. The organisms are also flagellated, and on account of a uglalike form which is present, de Korte ventures the suggestion that they belong to the class *ciliata*. In addition to being found in the blood, these bodies were also isolated from the chancre and the condyloma, but with considerable difficulty."

. Thus have we been furnished with different scientific discoveries of the causative germ, bacterium, protozoon or other microscopic originator of syphilis. Every one is correct, in his own estimation, and the mere looker-on in Venice is driven to his wits' end to make a rational choice or come to an intelligent conclusion. He is driven from pillar to post by the scientific men, and the practical, useful, indubitable and certain which he has sought is held off from him every time that he thinks he can grasp it. He is made to play the part of Tantalus, and finally, weary of the whole matter, he exclaims: "Under which king, Benzonian?"—*American Journal of Dermatology*.

---

### INTESTINAL ORIGIN OF PULMONARY TUBERCULOSIS.

In this third communication Chalmette (*Annals de l'Institut Pasteur*) states emphatically that any errors due to accidental inhalation of the microbes or insoluble particles were most scrupulously guarded against. His technique has been reviewed editorially in *The Journal*, October 6, 1906, page 1100. The animals were fed once with virulent tubercle bacilli, and the bacilli were refound in the lungs as early as the twenty-fourth hour in the adult animals, but not until the fifth day in the younger ones. The bacilli pass through the intercellular spaces in the intestinal epithelium without leaving a trace of their passage. As soon as they enter the chyle vessels they are incorporated by leucocytes and these transport them to the regional lymph nodes, where they are retained a longer or shorter time in proportion to the youth of the animal. In the very young animals they accumulate in the cortical layer of the lymph node, and if the amount of bacilli ingested was not very large, they remain there and produce tuberculous lesions. If only a small amount of bacilli are injested, the lymph nodes retain them, increase in size and allow a few—still incorporated in the leucocytes—to escape into the thoracic duct, and finally the nodes subside to normal size again. Inoculation of these glands in guinea-pigs shows that for a few months they still contain a small number of bacilli. If a considerable quantity of bacilli are ingested, small translucid tubercles appear throughout the lungs, but especially at the apices, on the anterior margins, and the pleural aspect, resembling in every respect fresh glanders tubercles in the horse. After a calf has had the single infecting meal, he reacts to tuberculin by the twenty-fifth to the thirtieth day. The tuberculous lesions are then established ; but, as a rule, they gradually heal, and after three or four months the tuberculin reaction no longer occurs and inoculation of the fibrous subpleural cicatricial tissues in the small cicatrices left no longer infects a guinea-pig. In adult animals there is no reaction on the part of the regional lymph nodes and the pulmonary lesions become established at once. Calmette is convinced that in the immense majority of cases, with the exception only of those in which bacilli were inhaled directly on some pre-existing lesion of the larynx or trachea, the pleural and pulmonary localizations of tuberculosis result from the arrest in the capillaries of the lungs or pleura of microphagic leucocytes immobilized by the toxic secretions of the bacilli which they have taken up. The same can probably be said also of all the localizations of tuberculosis in the bones, joints, meninges, etc. Glandular tuberculosis, he adds, is of intestinal origin. Its frequency in the young is explained by the special histologic characteristics of the lymphatics at this age. Animals, healing spontaneously, so that they cease to react to tuberculin after a few months, are no longer susceptible to infection, and can tolerate ingestion of comparatively large amounts of tubercle bacilli, at least for a certain period. They are thus vaccinated. On the other hand, animals given a second and third infecting meal, before they have entirely recovered from the first infection, never become cured. Their lesions continue always a progressive course. This explains why men and animals so often present entirely healed tuberculous lesions. They became infected with tuberculosis and had time to have their lesions heal before a second infection occurred, and thus they became vaccinated. Research on this aspect of vaccination is now in progress.—*Journal A. M. A.*, November 10, 1906.

---

### "AGREEMENTS AND SCHEDULE OF FEES."

When the Warren County Medical Society, at its October meeting, considered the question of fees for life-insurance examinations, it found itself opposed by an obstacle that, though removable, necessarily caused some delay; and since this same obstacle probably presents itself in many other counties, not only in Mississippi, but throughout the country, it seems advisable that attention be called to it.

Chapter II, Section 3, of the by-laws for county societies, as "prepared by the committee on organization of the American Medical Association"—which by-laws have, with slight alterations, been adopted everywhere—is as follows:

"Agreements and schedules of fees shall not be made by this society, but at least one meeting during each year shall be set apart for a discussion of the business affairs of the profession of the county, with the view of adopting best methods for the guidance of all. In all proper ways the public shall be taught that business methods and prompt collections are essential to the equipment of the modern physician and surgeon, and that it suffers even more than the profession when this is not recognized."

Adoption of "agreements and schedules of fees" by societies that have already subscribed to this section will obviously lead to friction in many instances, as there will be a loophole for escape on the ground of unconstitutionality. We therefore suggest that each society scan its by-laws carefully and make the necessary alterations where such have not been already made. The Warren County Society has now pending a motion to amend by striking out the first thirteen words of the above section, an alteration that seems to fit perfectly.

We do not know what construction the committee on organization of the American Medical Association puts on this section, but it appears to us that it must have been asleep during a good part of the Boston meeting.—*Mississippi Medical Monthly, November*, '*06*.

---

### FINAL RECOMMENDATIONS.

The Illinois State Board of Health now recommends formaldehyde as an aerial disinfectant, when used in combination with potassium permanganate.

The following quantities of chemicals should be used for each 1,000 cubic feet of air-space:

Temperature above 60° F.—Formaldehyde (40%), 16 ounces.
Potassium permanganate 6¾ ounces.
Temperature below 60° F.—Formaldehyde (40%) 24 ounces.
Potassium permanganate 10 ounces.

The following suggestions are important and may prevent failure in the application of this method of disinfection.

Good formaldehyde is essential to success. Reliable formaldehyde or formalin is not very expensive; poor formaldehyde is dear at any price. Get the best!

The permanganate of potassium must be in powdered form, or in long needle-shaped crystals. If the large octahedral crystals are purchased, they must be powdered before use. Get the best!

The retention of the heat caused by the reaction of the combined chemicals is necessary to the generation of a large volume of gas. Hence it is necessary that the metal container or generator be covered with asbestos, or with a non-conductive outer vessel.

As the union between the chemicals causes much frothing and effervesence, large vessels are necessary to prevent the solution from running over. The amounts of the chemicals set forth on page 21 should never be exceeded in vessels of the size mentioned.

The formaldehyde solution must be poured upon the potassium permanganate. The potassium permanganate must *not* be dropped in the formaldehyde.

The room should be sealed so as to prevent the escape of gas.

Clothing, bedding, etc., must be treated as described.

It is always well to wash the wood surfaces of the room, as described. It is absolutely necessary to do so when such surfaces have been soiled by the sputum or any other discharges of the patient.

It is *not* practicable to disinfect books with formaldehyde.

In conclusion, the State Board of Health earnestly advises and urges health officials, physicians and all others who may be called upon to perform disinfection, to adopt the method of aerial disinfection recommended herein, and to abandon the use of all other methods.—*Circular issued by the Illinois Board of Health.*

---

### THE PREVENTION OF CANCER.

Keetley believes that the grounds for attributing cancer to some living organism are exceedingly strong. He thinks that the time has come when we can lay down the following rules for the prevention of cancer: 1. Sterilize the food. The majority of cancers attack the alimentary canal, and especially the parts where food and feces tarry—e. g., the lips, the tongue, the tonsils, the esophagus, the lesser curvature of the stomach, the pylorus, the ileocecal region, the sigmoid flexure, the rectum, and the lower part of the large bowel. 2. The sufficient and regular toilet, and protection of the nipples and genitalia. These organs, so frequently attacked by cancer, are specially often polluted by stale secretions and discharges, and are more often handled than any other part of the body usually covered by clothing. During lactation special attention should be paid to cleanliness and dryness of the nipple, and to cleanliness of the infant's mouth. 3. Due care of the mouth and teeth. 4. The dressings of discharging malignant ulcerations should be destroyed carefully and not allowed to pollute either the fingers or the under linen. 5. Non-malignant sores and tumors should be cured and especially not be allowed to drift on if chronic. 6. As a matter of course, cancers and doubtful tumors and ulcers should be excised promptly. Early removal of a cancer performed in a correct and thorough manner not only gives the patient an excellent prospect of complete cure, but also removes a possible focus from which other people may acquire cancer. 7. Abstinence should be practiced from alcohol, tobacco, and from foods which leave waste products, of which the kidneys, the bowels, and the skin can not easily and thoroughly get rid, and which thereby provoke and sustain the chronic inflammations and ulcers which so often pave the way for cancer. Excess of meat eating should be strictly avoided. 8. Physical familiarity should be avoided, except with those who are nearest and dearest to us. 9. Much thought should be given to the service as well as to the cooking of food. Cooks and kitchen maids should be provided with all reasonable facilities for keeping their persons clean and their utensils aseptic, and should be regularly observed at their work in order to see if they have contracted aseptic habits. Special attention should be paid to the sterilization of milk and its products; milk comes in close contact with the skin, is analagous to the secretions of the sweat glands, and finally comes from just that portion of the outside of the body which is most prone to cancer.—*The Lancet*, *October 13*, *'06*.

---

### SUCCESS, THE SURGICAL DESIDERATUM.

Gallant lays especial stress on the following factors, which, when properly applied in appropriate cases, he believes, will ofttimes bring success and cure the patient:

A most careful consideration of antenatal and postnatal diseases and the chronicity and acuteness of the present condition before deciding to operate or not to operate.

The liberal use of water by mouth and rectum before, during and after operation. The free application of tincture of green soap, rubbed into the skin until dry, when preparing the field of operation and the hands of the operator and assistant.

Minimum morphin before anesthesia; care that the head is properly elevated; the ether administered by the patient herself in the first stage, and a minimum quantity of ether employed.

A reasonably short incision, a reasonably quick operation, a reasonable number of sutures to close the wound, and a reasonable time in bed.

After operation the careful prevention of chilling the body; the cautious use of morphin and sedatives; lavage to allay undue vomiting, and colonic massage and kneading for relief of distressing intestinal distention, with continuous rectal irrigation for tympany.

To invigorate the tired patient and to prevent muscular atrophy and cardiac weakness, general massage, with passive and active movements daily while in bed and calisthenics when first about.

For the prevention of hernia and enteroptosis or support of prolapsed organs apply the abdominal "stock." Rose's plaster bandage, or a special corset, put on while the patient is in the dorsal-inclined posture.—*A. Ernest Gallant, New York, Journal American Medical Association, October 17, '06.*

---

### SUGGESTIONS IN THE TREATMENT OF DISEASES OF THE RESPIRATORY TRACT.

BY J. F. T. JENKINS, PH.G., M.S., M.D., Los Angeles, Cal.

A marked advance in therapeutics has taken place in the last few years, and many new remedies have been introduced which, after careful clinical tests, have been found to be vastly superior to former methods of treatment. A drug which has attracted considerable attention is the new morphine derivative heroin. It has been brought before the profession for the purpose of allaying cough and to take the place of codeine as a more efficient substitute. Its action in relieving cough and dyspnea is much more prompt and decided, and the frequent deleterious after-effects of codeine and morphine—nausea, vomiting, headache, constipation, gastric pains, tinnitus and visual hallucinations—have never been observed during its administration. Results are equally as good with children as with adults, and it has now taken a permanent place in the armamentarium of the physician.

Some time after the introduction of heroin, while I was acting assistant surgeon in temporary command of the station for the United States Marine Hospital service, I had a number of government patients, sailors of the merchant marine, at the Los Angeles Infirmary (Sister's Hospital) under my professional care. In one case of persistent cough, which was extremely distressing to the patient and harassing to his friends, I tried everything I had heretofore used without obtaining even partial relief. The then resident physician, Dr. M. M. Kannon, urged me to try a new combination of heroin with other drugs, known as Glyco-Heroin (Smith), made by Martin H. Smith Company of New York. Acting upon this suggestion, I gave a prescription for a few ounces of this preparation to be given in teaspoonful doses every four to six hours until relieved. The good effect was immediate and pronounced, and from that time to the present I have had positive results in relieving cough that I had failed to obtain in my previous experience of a quarter of a century in the active practice of the medical profession. Before giving it in the case mentioned I was inclined to be sceptical, notwithstanding the frequency of favorable reports in regard to it by respectable medical journals and leading professional men. I had tried without satisfactory results the usual mixtures, "Heroin Comp.," of which there are so many without merit, failing entirely to accomplish all that heroin can be shown to do in using the preparation indicated. To satisfy myself still further and to remove a doubt in my mind that this might be an exceptional case or a mere coincidence, I was induced to give it a trial in a series of selected cases of a similar character. The result was so satisfactory that I feel constrained to add my testimony to that of others and place the record of cases before the profession; first giving a brief prelude dealing with the component parts of the prescription and its applicability in the treatment of all pulmonary and laryngeal diseases, followed by the expressed opinions of some eminent medical men as to the curative action of heroin, the most important drug in this excellent combination.

Glyco-Heroin (Smith) is a true solution of heroin in glycerine; each teaspoonful represents one sixteenth of a grain of heroin, with ammonium hypophosphite, hyoscyamus, white pine bark, balsam of tolu, with glycerine and aromatics. A glance at this formula shows a happy selection of drugs adding to the palliative effect of the heroin and each possessing decided curative properties of its own. It gives the physician an elegant pharmaceutical preparation, strictly ethical in character, a trial of which will satisfy him that it excels any single drug or combination of drugs in the materia medica. Limitation of space prevents an exhaustive consideration of the individual therapeutic virtues of the ingredients mentioned. With the exception of heroin, all are so well known that a very minute detail of their virtues is not necessary. It is important, however, to notice that the value of each seems to be increased tenfold, and the special sedative action intensified in the uniformly exact proportions adhered to by the Martin H. Smith Company in its manufacture. Referring again to the first component and most essential drug in this prescription—heroin—a recent writer says: "Though of recent date, this drug has already evinced its pronounced efficacy as a bronchial and pulmonary sedative and as an agent facilitating and promoting expectoration."

In the Boston *Medical and Surgical Journal*, February 22, 1900, Dr. Geis pronounces heroin "an eminently satisfactory agent in loosening and expelling stagnant products from the lungs."

Dr. H. D. Fulton, in the *New York Medical Journal*, refers to it as a "veritable boon to the consumptive as a palliative in cough."

In the *Jourual of the American Medical Association*, Dr. W. Freudenthal states that the "irritable cough in tuberculous patients frequently yields to the administration of heroin, and this occurs in cases in which both morphine and codeine have proved inefficient." The same writer considers it useful also in asthma and pseudo-croup.

W. H. Ambrose, M.D., of Cincinnati, O., reports as follows: "An agent like it, which diminishes the frequency of respiration and increases its force, finds a large field of application in conditions of dyspnea, as in nervous asthma and difficulty of breathing in cases of emphysema, chronic bronchitis, and especially phthisis."

Dr. E. R. Mitchell believes "it may be advantageously given in the cough and dyspnea of tuberculosis over a long period of time," while Dr. Morris Manges found it "a valuable aid in the treatment of pneumonia, alleviating the pleuritic stitches and the distressing cough. . . . It acted well in some cases not relieved by codeine."

A number of European authorities might also be quoted, notably Professor Leo, who speaks of it in a German medical publication as a "specific in affections of the bronchi attended with dyspnea and in emphysema."

So much for heroin, with a great deal left unsaid which might be justly stated in its favor. The ammonia hypophosphite and hyoscyamus each speaks for itself. The peculiar virtues of white pine bark in checking night sweats and in allaying all inflammatory conditions of the bronchial mucous membrane need only to be mentioned to be appreciated. That pleasant and palatable aromatic stimulant, balsam of colu, together with glycerine, completes the prescription known as Glyco-Heroin (Smith), now so well established by the evidence of experience.

The clinical reports of many practitioners in widely separated parts of the world attest this combination of remedies will quiet cough, remove dyspnea, relieve pain, aid and regulate expectoration, control night sweats, and cause the worn-out pulmonary invalid to add to his weight and build up his general health.

The clinical evidence as shown by these carefully-kept records has thus demonstrated that this preparation possesses qualities of unusual merit, that it contains elements within it calculated to reach the morbid state in which the symptoms originate and to correct the predisposition to further trouble.

I have used Glyco-Heroin (Smith) in the treatment of all disorders of the breathing apparatus, including coughs, colds, phthisis, laryngitis, asthma, bronchitis, pneumonia and whooping-cough. The following history of cases will give some idea of its wide range of usefulness.

In addition to my personal experience, I have reports from Dr. L. T. Holland, late coroner of Los Angeles county, and from Dr. Joseph Jauch, of this city, formerly of the University of Wurzburg, Germany.

Dr. Holland says he prescribes Glyco-Heroin (Smith) in preference to every other preparation of a similar nature, particularly for the relief of cough in its various forms. He speaks of its innocuous but powerful analgesic and antispasmodic properties. He closes a brief account of the good results he has obtained by stating that it has more than fulfilled his expectations.

Dr. Jauch states that he has at last found a valuable mixture that combines the two important elements of palatability and effectiveness. He has found it agrees perfectly with the most sensitive stomach. He continues: "I have found it the most efficient agent I have yet used in subduing the hyperesthesia of the respiratory mucous membrane. I have used it successfully in the treatment of most affections of the air passages, particularly for the relief of cough, obstinate cases of bronchitis, in severe laryngeal cough, and in many distressing cases of tubercular cough of an aggravated character which has resisted all other medication."

The striking and surprisingly good results so uniformly obtained in the administration of this remedy can be fully verified by any unprejudiced trial in which it is tested. Such a trial may be made without hesitation, for notwithstanding its therapeutic advantages it possesses the virtue of absolute harmlessness. When physicians of the professional standing of Francis W. Campbell, M.A., M.D., D.C.L., L.R.C.P., London, dean and professor of medicine, University of Bishop's College, Montreal; and Dr. J. Leffingwell Hatch, late professor of laryngology in the New York Clinical School of Medicine, pathologist to the Philadelphia Hospital and formerly sanitary inspector in the

Marine Hospital Service, give this preparation their unqualified endorsement, their opinions founded on the actual treatment of a large number of cases, it is apparent that these positive, unlimited and clear results must gain for this remedy a still fuller recognition, and lead ultimately to its universal acknowledgment as the best remedy of its class for the purposes indicated in these reports and in clinical reports of prominent medical men in England and her colonies, in addition to the favorable testimony of many American physicians from Canada to Mexico and from Maine to California.

---

URETHRAL BACTERIOLOGY IN THE ETIOLOGY OF CYSTITIS.

Taussig summarizes the relationship of urethral bacteria to the production of catheter cystitis in women as follows: The normal urethra, free of disease, is sterile in only a small proportion of cases. Pathogenic germs are present in about half of the total number of urethræ. Of the pathogenic bacteria found staphylococcus pyogenes albus is the most common. The colon bacillus is frequently found where patients are confined to bed. That these pathogenic bacteria are actually carried into the bladder by catheterization was shown in eight examinations in which the urethral secretion and the urine obtained by catheterization were compared. Irrigation of the urethra with boric acid removes many of the urethral bacteria, but not all of them when their number is great. The double catheters thus far devised to avoid contamination with the urethral bacteria are not as satisfactory as the ordinary glass catheter. When repeated catheterization becomes necessary precautions must be taken to prevent infection. Urotropin should be administered, and before each catheterization a glass catheter should be inserted part way, the urethra irrigated with half a pint of boric acid solution, then introduced into the bladder, the latter evacuated and then irrigated with one or two pints additional of boric acid solution.—*American Journal of Obstetrics*, *October*, *1906*.

---

IN performing operations on the neck, make the skin incision parallel to the muscular plane.—*American Journal of Surgery*.

### JUST HOW TO MANAGE OTORRHEA.

BY F. E. BERGEVIN, M.D., OF SPIRO, I. T.

(Published by *The Kansas City Medical Record*, July, 1906.)

Otorrhea, from purulent middle-ear catarrh, the "running ears" of the laity, was at first a *bete noire*. I used the classic treatment of Pomeroy and others—syringing, insufflations of powdered boric acid, etc., sometimes with *benefit*, sometimes the reverse, but never by any chance *curing* any of them, until I dreaded to see a patient with cotton in his ears come into the office. Now I cure them in a few days or weeks without difficulty, and really prefer this class of cases to any other. When I was at the Manhattan Eye and Ear Hospital in 1890, Dr. Pomeroy said that one case had been under treatment nearly ten months and was *slightly improved*. He said that it required one or two years to cure this disease, and then it generally returned.

My method of treatment is as simple as it is effectual, and any doctor after reading my description attentively can use it as well as I can and cure every case. Once daily I fill the ear with a warm solution of some good peroxide of hydrogen, beginning with a twenty-five per cent. solution, and increasing the strength every day until the pure drug is used. Hydrozone is the same, only twice as strong, and I use it when I can get it simply from motives of economy. After cleansing the ear thoroughly, which at first may require from twenty minutes to two hours, according to the foulness of the auditory canal, I then instill a few drops of Glycozone (warmed) and close the canal securely with a bit of absorbent cotton. This is allowed to remain *in situ* until the next treatment.

The *cleansing* should be very thorough, the peroxide being repeatedly instilled until all foaming ceases. In some cases it may require two, three or more treatments to cleanse the ear properly, especially if the lumen be occluded by a furuncle, or by swelling, or inspissated discharge. Do not be discouraged by any little difficulty like this, keep right on and you will finally succeed in getting the ear *clean*. After that it is plain sailing.

Thenceforth the daily treatment need not consume more than ten to twenty minutes. It is better to treat the case every day, but I have had good success with patients who could not come oftener than once a week. Do not give the patient medicine to use at home and expect to cure him; and never tell him what you are using.

In children who dread the procedure, I do not attempt much the first time or two, but strive to win their confidence, which is not ordinarily difficult, as the treatment is not at all painful and is always followed by a certain sense of relief, so that children who are in mortal terror of me at first will, after a few treatments, come to me of their own accord. Even babies of one and two years who would not suffer me to touch them at first, after experiencing the grateful relief afforded, will place the head on the chair in the proper position and gladly submit to the treatment.

When the diseased ear has been thoroughly cleansed I consider my work as half done. Thenceforth improvement is usually very rapid, even old inveterate cases yielding in a few weeks. Relapses occur, but are easily managed, and I have seldom had a second relapse. Of course, mastoid disease, necrosis, polyps, etc., must receive appropriate treatment; but I have no hesitation in saying that all simple, uncomplicated cases (which include the vast majority of all cases under one year's duration) may be cured by this treatment if it is properly and thoroughly carried out.

Care must be taken to have the medicaments *warm* and not too hot—100 F. is about right—and to always stop up the ear with a bit of aseptic cotton before permitting the patient to leave the office. Be careful to use a piece of cotton of just the right size to securely close up the meatus; if too large it will work out, allowing the solution to escape and leaving the ear unprotected; if too small it will slip back into the canal and so fail of its effect.

Never syringe the ears in otorrhea; it is risky and useless. I usually drop a little warm solution of sodium borate—5 per cent.—in the ear to prevent a slight stinging which sometimes ensues when active steps are taken. I also dry out the canal with cotton on an applicator, but this should be very carefully done with speculum, and the canal well lighted. These points are non-essentials, merely refinements which render the treatment a trifle more pleasant, perhaps; that is about all.

The general health will probably require overhauling, indications being met as they arise. It is a good idea to regulate and antisepticise the bowels as a routine measure, using salines and intestinal antiseptics—*e. g.*, the sulphocarbolates as needed. In the South especially, malarial and other miasmatic affections will often need looking after; also any other existing disease may require attention, but it is presumed that the practician will know how to handle these.

We all ought to try to help each other; not one of us but has much to learn; and in these brief talks it is my principal aim to set an example for the rest, hoping my little crumbs may some day come back to me in the shape of seasonable aid from some brother who has a few pointers himself and, like me, is willing to "whack up."

---

### TREATMENT OF CANCER OF THE RECTUM.

Du Pan concludes his paper as follows: Heredity, sedentary occupation, habitual constipation, hemorrhoids, and traumatisms do not have the etiological influence that has generally been ascribed to them. Cancer of the rectum develops more rapidly prior to the fortieth year than at later periods of life, and usually takes the colloid form at that period. Its prognosis is bad. Its insidious beginning is one of the chief obstacles to the improvement in the ultimate result of operative measures, an early diagnosis being of rare occurrence. This shows the importance of rectal examination in all rectal disorders. The average duration of the disease in nonoperated cases is twelve months from the beginning of the symptoms. In operated cases the average duration is fifty-six months. Immobility of the tumor is the sole contraindication to a radical operation. Preliminary colostomy should be avoided if possible.

Only in cases of severe ileus should it be practiced. The coccygeal operation of Kocher should not be confused with the perineal and sacral methods. Kocher's method is recommended as the most effective and desirable. The diseased segment having been removed the end of the intestine should be secured to the anus, the mucous membrane of the latter having been dissected away. The peritoneum should be carefully sutured and not tamponed or drained. The aim of the subsequent treatment should be the avoidance of infection. Should infection appear, irrigation and drainage should be rigorously carried out. The operative mortality with the author has been 14.58 per cent. Ether should be used as the anesthetic. Recurrence took place in 39.7 per cent. of his cases, usually in the circumrectal tissue.—*Revue de Chirurgie*, *August*, *1906*.

---

### SOME REMARKS ON THE DIET OF CHILDHOOD.

Dr. D. J. M. Miller (in the *Therapeutic Gazette*, September, 1906) takes up the feeding of healthy children and says: "In advising a liberal diet for children, I am fully aware that the tendency of people to err in this direction is greater than the reverse—that they need restraining more than urging, epecially the parents of the mechanic and poorer class, who are apt to feed their offspring food unsuitable both in character and in the method of preparation. Particularly harmful is the practice, so prevalent among this class, of permitting even the youngest children to drink strong tea and coffee, and sometimes beer. At the same time I am convinced that it is to the child's present and future physical and moral well-being that it be taught to eat every article of wholesome food; and that much of the opprobrium attached to many articles of diet is, in reality, due to eating between meals, to improper preparation, and to hurried eating and overeating. Finally, it is well to remember that in feeding a child the simplest and most digestible meats and vegetables should first be given; then, as the child grows older, other wholesome articles should be successively added, until the ordinary diet of adults of its class and station is arrived at. Further, it should be borne in mind that the stomach of the child is an organ that does not differ from the adult's in that it grows strong with the using and weak with the non-using—that it literally grows by what it feeds on; and, still further, that individualization is often an essential factor in the successful nourishment of children."

---

### THE SURGICAL TREAMENT OF TRIGEMINAL NEURALGIA.

Moschcowitz concludes his observation in saying that the treatment of trigeminal neuralgia may be summed up as follows: Eliminate any possible etiological factors, such as tumors, carious teeth, antral disease, malaria, syphilis, etc. Determine accurately the nerve branch or branches involved. The operation should be performed as near to the periphery as possible. The operation should be performed early. This is important, because the earlier the case the more chances there are that a peripheral operation will be of benefit. Whatever the character of the operation may be, the dominant principle must be the prevention of regeneration of the affected nerve. More specifically, the operations may be classed under two headings, depending on the nerve or nerves affected: (a) Peripheral operations. If the supraorbital, infraorbital, mental, malar, or inferior dental branches, either singly or collectively, are involved, the operation consists in division of the nerve, and plugging up of the foramen by a gold or silver button or wire. (b) Central operations. If the neuralgia involves the upper teeth and palate (superior maxillary division) or tongue (inferior maxillary division) existing either singly or together with the other nerves mentioned, the operation as has been outlined by Abbe must always be performed, substituting, however, celluloid or a gold button instead of rubber tissue. Finally, he believes that if these stated principles of treatment of trigeminal neuralgia are carried out, the operation of extirpation of the Gasserian ganglion will become entirely unnecessary.—*Medical Record*, *September 29*, *'06*.

